# Supplementary material for: Profiling of hMPV F-specific antibodies isolated from human memory B cells
Source: Nat Commun. 2022 May 10;13:2546. doi: 10.1038/s41467-022-30205-x (PMC9091222; doi:10.1038/s41467-022-30205-x)
Supplement: Supplementary file 1 — Supplementary Information [file 41467_2022_30205_MOESM1_ESM.pdf]

Profiling of hMPV F-specific antibodies isolated from human memory B cells

Xiao Xiao[1,2,3], Arthur Fridman[4], Lu Zhang[5], Pavlo Pristatsky[6], Eberhard Durr[1], Michael Minnier[7], Aimin Tang[1], Kara S. Cox[1], Zhiyun Wen[1], Renee Moore[2], Dongrui Tian[8], Jennifer D. Galli[1], Scott Cosmi[9], Michael J. Eddins[10], Nicole L. Sullivan[1], Xiaodong Yan[8], Andrew J. Bett[1], Hua-Poo Su[10], Kalpit A. Vora[1*], Zhifeng Chen[1*], Lan Zhang[1*]

[1] Infectious Diseases and Vaccines Discovery, Merck & Co., Inc., West Point, PA, USA

[2] Discovery Biologics, Merck & Co., Inc., Boston, Massachusetts, USA

[3] MRL Postdoctoral Research Program; Merck & Co., Inc., Kenilworth, NJ, USA

[4] Data Science and Scientific Informatics, Merck & Co., Inc., Rahway, NJ, USA

[5] Bioinformatics and Biomarker Research, MSD, Beijing, China

[6] Analytical Research and Development, Merck & Co., Inc., West Point, PA, USA

[7] AgileOne, Torrence, CA, USA

[8] Wuxi Biortus Biosciences Co. Ltd., Wuxi, China

[9] Eurofins PSS Insourcing Solutions, Lancaster, PA, USA

[10] Computational and Structural Chemistry, Merck & Co., Inc., West Point, PA, USA

* Corresponding authors

Email: kalpit.vora@merck.com (KV), zhifeng.chen@merck.com (ZC), lan_zhang2@merck.com (LZ)

The materials include content:

Supplementary Tables 1 to 2

Supplementary Figure 1 to 13

Supplementary Table 1. Raw data of the individual ELISA titers in serum absorption assays

ELISA binding antigens	Absorb antigen	ELISA titer (fold dilution)			
		#84974	#86559	#21083	#85359
Unprocessed hMPV WT PreF trimer	Unprocessed hMPV WT PreF trimer	<1	<1	<1	<1
Processed stabilized hMPV PreF trimer (115BV)		<1	377	<1	<1
hMPV PostF trimer		<1	<1	<1	<1
RSV PreF (DS-Cav1)		3423	5508	3975	6039
Unprocessed hMPV WT PreF trimer	Processed stabilized hMPV PreF trimer (115BV)	<1	144	656	<1
Processed stabilized hMPV PreF trimer (115BV)		<1	<1	521	<1
hMPV PostF trimer		<1	41	250	<1
RSV PreF (DS-Cav1)		4504	2750	4771	6141
Unprocessed hMPV WT PreF trimer	MPV PostF (dPFv1)	673	26378	582	22
Processed stabilized hMPV PreF trimer (115BV)		0	15595	311	<1
hMPV PostF trimer		56	1891	58	75
RSV PreF (DS-Cav1)		3306	4458	5293	5755
Unprocessed hMPV WT PreF trimer	RSV PreF (DS-Cav1)	5129	55865	5520	2428
Processed stabilized hMPV PreF trimer (115BV)		4103	36424	5350	1862
hMPV PostF trimer		5224	29467	4344	2957
RSV PreF (DS-Cav1)		<1	<1	<1	239
Unprocessed hMPV WT PreF trimer	PBS buffer	4989	56976	7154	3966
Processed stabilized hMPV PreF trimer (115BV)		4555	29594	5377	2753
hMPV PostF trimer		5107	25086	5279	4227
RSV PreF (DS-Cav1)		3281	4204	5359	5006

Supplementary Table 2. Primer sequences for NGS human B cell IgG cloning

**RT-PCR primers**			
		**Primer name**	**Primer sequence**
**Heavy chain**	5’ primer	5' LH1/7 For	5’ ACAGGTGCCCACTCCCAGGTGCAG
		5' LH2 For	5' CTGACCATCCCTTCATGGGTCTTGTCC
		5' LH3 For	5' AAGGTGTCCAGTGTGARGTGCAGCTG
		5' LH4/6 For	5' AGATGGGTCCTGTCCCAGGTGCAG
		5' LH5 For	5' CAAGGAGTCTGTTCCGAGGTGCAGC
	3’ primer	3' IgG CH2 Rev	5' GCTCACGTCCACCACCACG
**Kappa chain**	5’ primer	5' LK1/2 For	5' ATGAGGGTCCCYGCTCAGCTCCTG
		5' LK3 For	5' CTCTTCCTCCTGCTACTCTGGCTC
		5' LK4 For	5' CAGGTCTTCATTTCTCTGTTGCTCTGG
		5' LK5 For	5' GTTCACCTCCTCAGCTTCCTCCTC
		5' LK6 For	5' CTCTGGGTTCCAGCCTCCAGG
	3’ primer	3' CKappa Rev	5' ACACTCTCCCCTGTTGAAGCTCTTTGTG
**Lambda chain**	5’ primer	5' Lλ1 For	5' GGTCCTGGGCCCAGTCTGTGCTG
		5' Lλ2 For	5' GGTCCTGGGCYCAGTCTGCCCTG
		5' Lλ3 For	5' GCTCTGWGGCCTCCTATGAGCTG
		5' Lλ4,5,9 For	5' GGTCTCTCTCSCAGCYTGTGCTG
		5' Lλ6 For	5' GTTCTTGGGCCAATTTTATGCTGACTC
		5' Lλ7 For	5' GGTCCAATTCYCAGGCTGTGGTG
		5' Lλ8 For	5' GAGTGGATTCTCAGACTGTGGTG
		5' Lλ10 For	5' GTGTCAGTGGTCCAGGCAGGGCTG
	3’ primer	3' CLambda Rev	5' CATTCYGYAGGGGCMACTGTCTTC
**Nested-PCR primers**			
		**Primer name**	**Primer sequence**
**Heavy chain**	5’ primer	5' VH1,7 ovlp_For	5’ ACACTCTTTCCCTACACGACGCTCTTCCGATCTCAGGTGCAGCTGGTGCAGTCTGG
		5' VH2 ovlp_For	5’ ACACTCTTTCCCTACACGACGCTCTTCCGATCTCAGATCACCTTGAAGGAGTCTGG
		5' VH3,5 ovlp_For	5’ ACACTCTTTCCCTACACGACGCTCTTCCGATCTGAGGTGCAGCTGGTGSAGTCTGG
		5' VH4 ovlp_For	5’ ACACTCTTTCCCTACACGACGCTCTTCCGATCTCAGGTGCAGCTGCAGSAGTCGGG
		5' VH6 ovlp_For	5’ ACACTCTTTCCCTACACGACGCTCTTCCGATCTCAGGTACAGCTGCAGCAGTCAGG
	3’ primer	3' READ2-HR88	5' GTGACTGGAGTTCAGACGTGTGCTCTTCCGATCTTGACCAGGCAGCCCAGG
**Kappa chain**	5’ primer	5' VK1a, b ovlp_For	5’ ACACTCTTTCCCTACACGACGCTCTTCCGATCTGACATCCAGWTGACCCAG
		5' VK1c ovlp_For	5’ ACACTCTTTCCCTACACGACGCTCTTCCGATCTGCCATCCGGTTGACCCAG
		5' VK2 ovlp_For	5’ ACACTCTTTCCCTACACGACGCTCTTCCGATCTGATATTGTGATGACYCAG
		5' VK3a, b ovlp_For	5’ ACACTCTTTCCCTACACGACGCTCTTCCGATCTGAAATTGTGTTGACRCAG
		5' VK3c ovlp_For	5’ ACACTCTTTCCCTACACGACGCTCTTCCGATCTGAAATAGTGATGACGCAG
		5' VK4 ovlp_For	5’ ACACTCTTTCCCTACACGACGCTCTTCCGATCTGACATCGTGATGACCCAG
		5' VK5 ovlp_For	5’ ACACTCTTTCCCTACACGACGCTCTTCCGATCTGAAACGACACTCACGCAG
		5' VK6a ovlp_For	5’ ACACTCTTTCCCTACACGACGCTCTTCCGATCTGAAATTGTGCTGACTCAG
		5' VK6b ovlp_For	5’ ACACTCTTTCCCTACACGACGCTCTTCCGATCTGATGTTGTGATGACACAG
	3’ primer	3' READ2-KR26	5' GTGACTGGAGTTCAGACGTGTGCTCTTCCGATCTAAGACAGATGGTGCAGC
**Lambda chain**	5’ primer	5' VL1 ovlp_For	5’ ACACTCTTTCCCTACACGACGCTCTTCCGATCTCAGTCTGTGCTGACKCAG
		5' VL2 ovlp_For	5’ ACACTCTTTCCCTACACGACGCTCTTCCGATCTCAGTCTGCCCTGACTCAG
		5' VL3 ovlp_For	5’ ACACTCTTTCCCTACACGACGCTCTTCCGATCTTCCTATGAGCTGACWCAG
		5' VL4,5,9 ovlp_For	5’ ACACTCTTTCCCTACACGACGCTCTTCCGATCTCAGCYTGTGCTGACTCAG

		5' VL6 ovlp_For	5’ ACACTCTTTCCCTACACGACGCTCTTCCGATCTAATTTTATGCTGACTCAG
		5' VL7,8 ovlp_For	5’ ACACTCTTTCCCTACACGACGCTCTTCCGATCTCAGRCTGTGGTGACTCAG
		5' VL10 ovlp_For	5’ ACACTCTTTCCCTACACGACGCTCTTCCGATCTCAGGCAGGGCTGACTCAG
	3’ primer	3' READ2-LR42	5’ GTGACTGGAGTTCAGACGTGTGCTCTTCCGATCTGGGYGGGAACAGAGTGAC
**Barcoding-PCR primers**			
		**Primer name**	**Primer sequence**
**Heavy chain**	5’ primer (column)	P5-S502-READ1os	AATGATACGGCGACCACCGAGATCTACACCTCTCTATACACTCTTTCCCTACACGACGC
		P5-S503-READ1os	AATGATACGGCGACCACCGAGATCTACACTATCCTCTACACTCTTTCCCTACACGACGC
		P5-S505-READ1os	AATGATACGGCGACCACCGAGATCTACACGTAAGGAGACACTCTTTCCCTACACGACGC
		P5-S506-READ1os	AATGATACGGCGACCACCGAGATCTACACACTGCATAACACTCTTTCCCTACACGACGC
		P5-S507-READ1os	AATGATACGGCGACCACCGAGATCTACACAAGGAGTAACACTCTTTCCCTACACGACGC
		P5-S508-READ1os	AATGATACGGCGACCACCGAGATCTACACCTAAGCCTACACTCTTTCCCTACACGACGC
		P5-S510-READ1os	AATGATACGGCGACCACCGAGATCTACACCGTCTAATACACTCTTTCCCTACACGACGC
		P5-S511-READ1os	AATGATACGGCGACCACCGAGATCTACACTCTCTCCGACACTCTTTCCCTACACGACGC
	3’ primer (Row)	P7-N701-READ2os	CAAGCAGAAGACGGCATACGAGATTCGCCTTAGTGACTGGAGTTCAGACGTGTGC
		P7-N702-READ2os	CAAGCAGAAGACGGCATACGAGATCTAGTACGGTGACTGGAGTTCAGACGTGTGC
		P7-N703-READ2os	CAAGCAGAAGACGGCATACGAGATTTCTGCCTGTGACTGGAGTTCAGACGTGTGC
		P7-N704-READ2os	CAAGCAGAAGACGGCATACGAGATGCTCAGGAGTGACTGGAGTTCAGACGTGTGC
		P7-N705-READ2os	CAAGCAGAAGACGGCATACGAGATAGGAGTCCGTGACTGGAGTTCAGACGTGTGC
		P7-N706-READ2os	CAAGCAGAAGACGGCATACGAGATCATGCCTAGTGACTGGAGTTCAGACGTGTGC
		P7-N707-READ2os	CAAGCAGAAGACGGCATACGAGATGTAGAGAGGTGACTGGAGTTCAGACGTGTGC
		P7-N710-READ2os	CAAGCAGAAGACGGCATACGAGATCAGCCTCGGTGACTGGAGTTCAGACGTGTGC
		P7-N711-READ2os	CAAGCAGAAGACGGCATACGAGATTGCCTCTTGTGACTGGAGTTCAGACGTGTGC
		P7-N712-READ2os	CAAGCAGAAGACGGCATACGAGATTCCTCTACGTGACTGGAGTTCAGACGTGTGC
		P7-N714-READ2os	CAAGCAGAAGACGGCATACGAGATTCATGAGCGTGACTGGAGTTCAGACGTGTGC
		P7-N715-READ2os	CAAGCAGAAGACGGCATACGAGATCCTGAGATGTGACTGGAGTTCAGACGTGTGC
**Kappa chain**	5’ primer (column)	P5-S513-READ1os	AATGATACGGCGACCACCGAGATCTACACTCGACTAGACACTCTTTCCCTACACGACGC
		P5-S515-READ1os	AATGATACGGCGACCACCGAGATCTACACTTCTAGCTACACTCTTTCCCTACACGACGC
		P5-S516-READ1os	AATGATACGGCGACCACCGAGATCTACACCCTAGAGTACACTCTTTCCCTACACGACGC
		P5-S517-READ1os	AATGATACGGCGACCACCGAGATCTACACGCGTAAGAACACTCTTTCCCTACACGACGC
		P5-S518-READ1os	AATGATACGGCGACCACCGAGATCTACACCTATTAAGACACTCTTTCCCTACACGACGC
		P5-S520-READ1os	AATGATACGGCGACCACCGAGATCTACACAAGGCTATACACTCTTTCCCTACACGACGC
		P5-S521-READ1os	AATGATACGGCGACCACCGAGATCTACACGAGCCTTAACACTCTTTCCCTACACGACGC
		P5-S522-READ1os	AATGATACGGCGACCACCGAGATCTACACTTATGCGAACACTCTTTCCCTACACGACGC
	3’ primer (Row)	P7-N701-READ2os	CAAGCAGAAGACGGCATACGAGATTCGCCTTAGTGACTGGAGTTCAGACGTGTGC
		P7-N702-READ2os	CAAGCAGAAGACGGCATACGAGATCTAGTACGGTGACTGGAGTTCAGACGTGTGC
		P7-N703-READ2os	CAAGCAGAAGACGGCATACGAGATTTCTGCCTGTGACTGGAGTTCAGACGTGTGC
		P7-N704-READ2os	CAAGCAGAAGACGGCATACGAGATGCTCAGGAGTGACTGGAGTTCAGACGTGTGC
		P7-N705-READ2os	CAAGCAGAAGACGGCATACGAGATAGGAGTCCGTGACTGGAGTTCAGACGTGTGC
		P7-N706-READ2os	CAAGCAGAAGACGGCATACGAGATCATGCCTAGTGACTGGAGTTCAGACGTGTGC
		P7-N707-READ2os	CAAGCAGAAGACGGCATACGAGATGTAGAGAGGTGACTGGAGTTCAGACGTGTGC
		P7-N710-READ2os	CAAGCAGAAGACGGCATACGAGATCAGCCTCGGTGACTGGAGTTCAGACGTGTGC
		P7-N711-READ2os	CAAGCAGAAGACGGCATACGAGATTGCCTCTTGTGACTGGAGTTCAGACGTGTGC
		P7-N712-READ2os	CAAGCAGAAGACGGCATACGAGATTCCTCTACGTGACTGGAGTTCAGACGTGTGC
		P7-N714-READ2os	CAAGCAGAAGACGGCATACGAGATTCATGAGCGTGACTGGAGTTCAGACGTGTGC
		P7-N715-READ2os	CAAGCAGAAGACGGCATACGAGATCCTGAGATGTGACTGGAGTTCAGACGTGTGC
**Lambda chain**	5’ primer (column)	P5-S502-READ1os	AATGATACGGCGACCACCGAGATCTACACCTCTCTATACACTCTTTCCCTACACGACGC
		P5-S503-READ1os	AATGATACGGCGACCACCGAGATCTACACTATCCTCTACACTCTTTCCCTACACGACGC
		P5-S505-READ1os	AATGATACGGCGACCACCGAGATCTACACGTAAGGAGACACTCTTTCCCTACACGACGC

	P5-S506-READ1os	AATGATACGGCGACCACCGAGATCTACACACTGCATAACACTCTTTCCCTACACGACGC
	P5-S507-READ1os	AATGATACGGCGACCACCGAGATCTACACAAGGAGTAACACTCTTTCCCTACACGACGC
	P5-S508-READ1os	AATGATACGGCGACCACCGAGATCTACACCTAAGCCTACACTCTTTCCCTACACGACGC
	P5-S510-READ1os	AATGATACGGCGACCACCGAGATCTACACCGTCTAATACACTCTTTCCCTACACGACGC
	P5-S511-READ1os	AATGATACGGCGACCACCGAGATCTACACTCTCTCCGACACTCTTTCCCTACACGACGC
3’ primer (Row)	P7-N716-READ2os	CAAGCAGAAGACGGCATACGAGATTAGCGAGTGTGACTGGAGTTCAGACGTGTGC
	P7-N718-READ2os	CAAGCAGAAGACGGCATACGAGATGTAGCTCCGTGACTGGAGTTCAGACGTGTGC
	P7-N719-READ2os	CAAGCAGAAGACGGCATACGAGATTACTACGCGTGACTGGAGTTCAGACGTGTGC
	P7-N720-READ2os	CAAGCAGAAGACGGCATACGAGATAGGCTCCGGTGACTGGAGTTCAGACGTGTGC
	P7-N721-READ2os	CAAGCAGAAGACGGCATACGAGATGCAGCGTAGTGACTGGAGTTCAGACGTGTGC
	P7-N722-READ2os	CAAGCAGAAGACGGCATACGAGATCTGCGCATGTGACTGGAGTTCAGACGTGTGC
	P7-N723-READ2os	CAAGCAGAAGACGGCATACGAGATGAGCGCTAGTGACTGGAGTTCAGACGTGTGC
	P7-N724-READ2os	CAAGCAGAAGACGGCATACGAGATCGCTCAGTGTGACTGGAGTTCAGACGTGTGC
	P7-N726-READ2os	CAAGCAGAAGACGGCATACGAGATGTCTTAGGGTGACTGGAGTTCAGACGTGTGC
	P7-N727-READ2os	CAAGCAGAAGACGGCATACGAGATACTGATCGGTGACTGGAGTTCAGACGTGTGC
	P7-N728-READ2os	CAAGCAGAAGACGGCATACGAGATTAGCTGCAGTGACTGGAGTTCAGACGTGTGC
	P7-N729-READ2os	CAAGCAGAAGACGGCATACGAGATGACGTCGAGTGACTGGAGTTCAGACGTGTGC

K = G / T, M = A / C, R = A / G, S = G / C, W = A / T, Y = C / T, H = A / C / T.

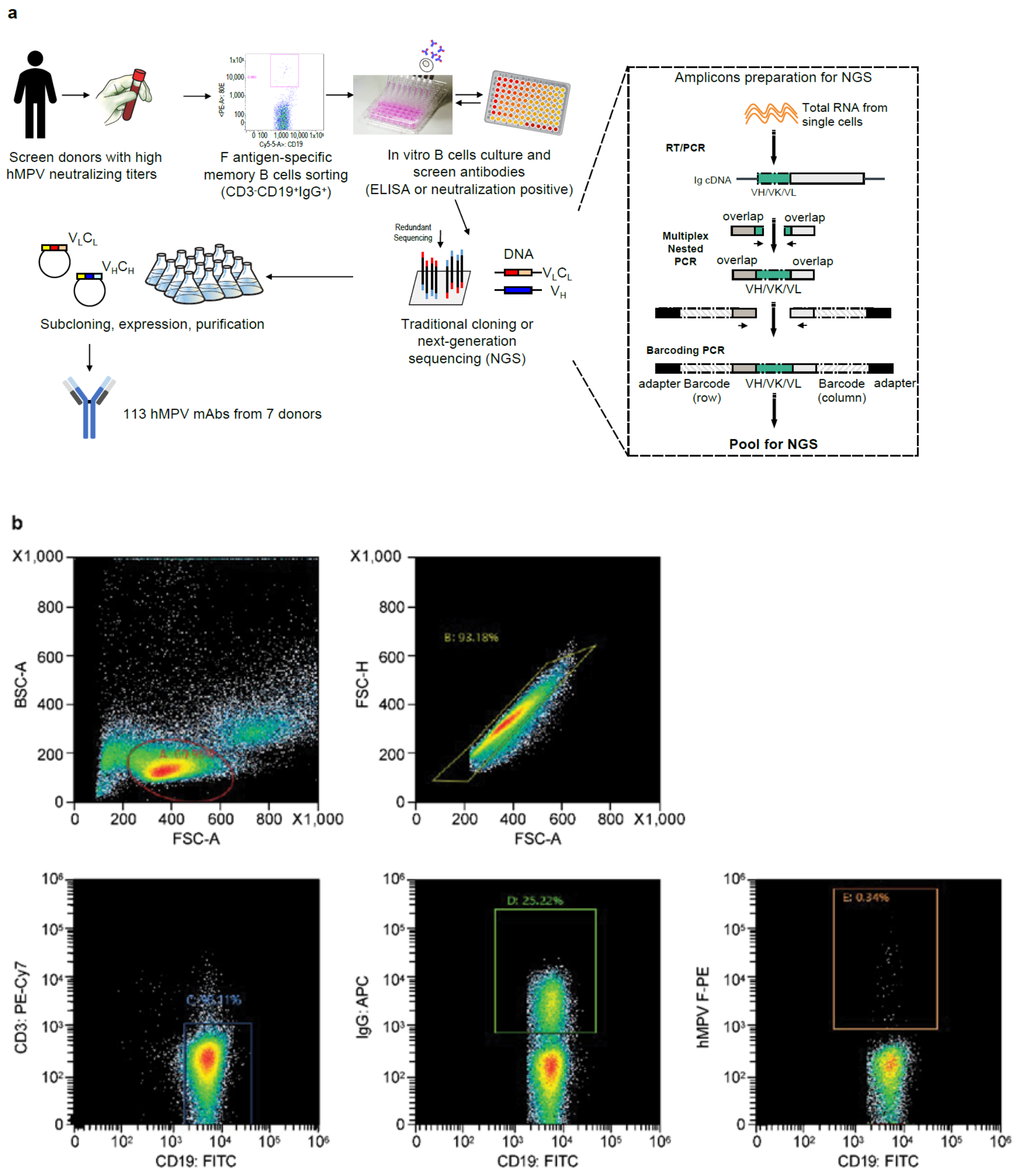
a
Screen donors with high hMPV neutralizing titers
F antigen-specific memory B cells sorting (CD3-CD19+IgG+)
In vitro B cells culture and screen antibodies (ELISA or neutralization positive)
Amplicons preparation for NGS
Total RNA from single cells
RT/PCR
Ig cDNA
VH/VK/VL
Multiplex Nested PCR
overlap
Barcoding PCR
adapter Barcode (row)
Barcode (column) adapter
Pool for NGS
Redundant Sequencing
DNA
Traditional cloning or next-generation sequencing (NGS)
Subcloning, expression, purification
113 hMPV mAbs from 7 donors
b
BSC-A
FSC-A
FSC-H
B: 93.18%
CD3: PE-Cy7
IgG: APC
hMPV F-PE
CD19: FITC
D: 25.22%
E: 0.34%

Supplementary Figure 1. HMPV-F specific antibody discovery by single memory B cell sorting, culturing and cloning. (**A**) Workflow of antibody discovery by single memory B cell sorting, culturing and cloning. (**B**) Representative B-cell sorting gating strategy. Enriched B-cells were gated using light Back Scatter (BSC) and Forward Scatter (FSC) followed by forward scatter height (FSC-H) by area to exclude debris and doublet cells. Next, cells were gated on CD3 followed by $CD19^+$ IgG^+ cells. These cells were then used to gate on antigen specific cells (hMPV F^- PE^+).

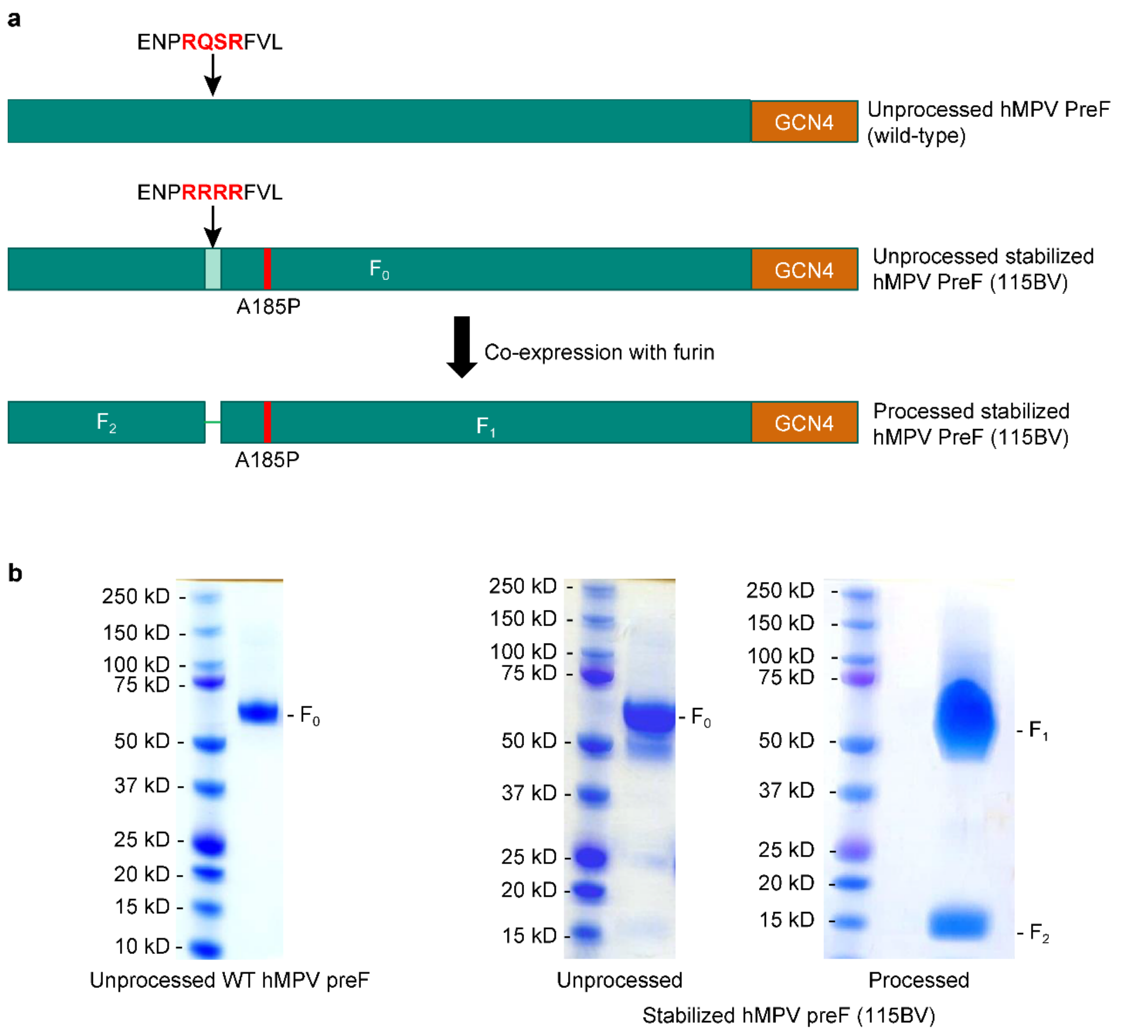

Supplementary Figure 2. Schematic diagram of hMPV PreF constructs (A) and purified PreF proteins on denatured SDS-PAGE gels (B). Each experiment was repeated independently at least twice with similar results, and representative gels are shown.

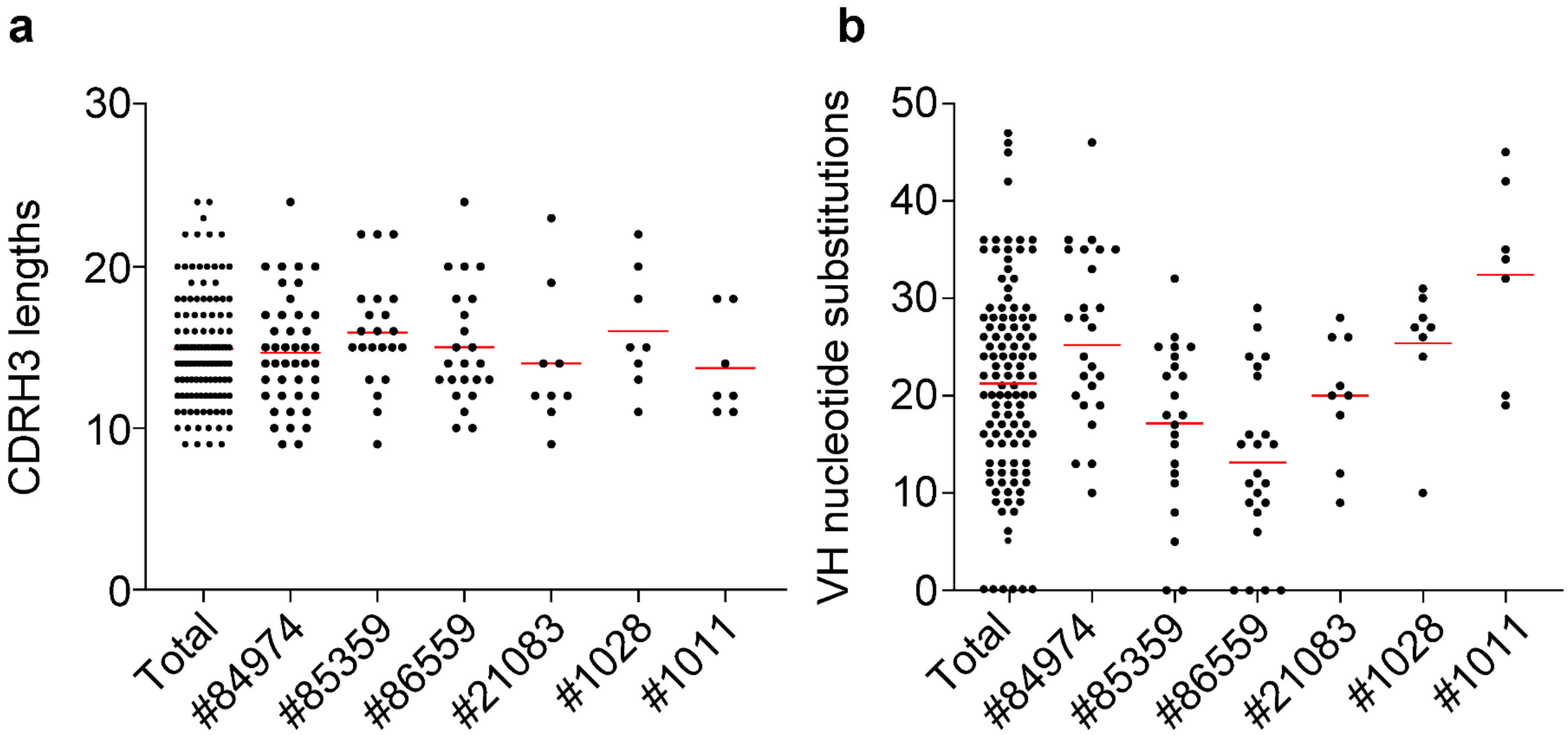

Supplementary Figure 3. CDR H3 lengths and Somatic hypermutations (SHM) of isolated mAbs, grouped by donors. (**A**) CDR H3 lengths. (**B**) Nucleotide substitutions of VH (excluding CDRH3). Red bars indicate the median. Donor #BSC2 was not included as only one mAb was isolated from this donor. The analysis was based on Kabat delineation system [75]. Source data are provided as a Source Data file.

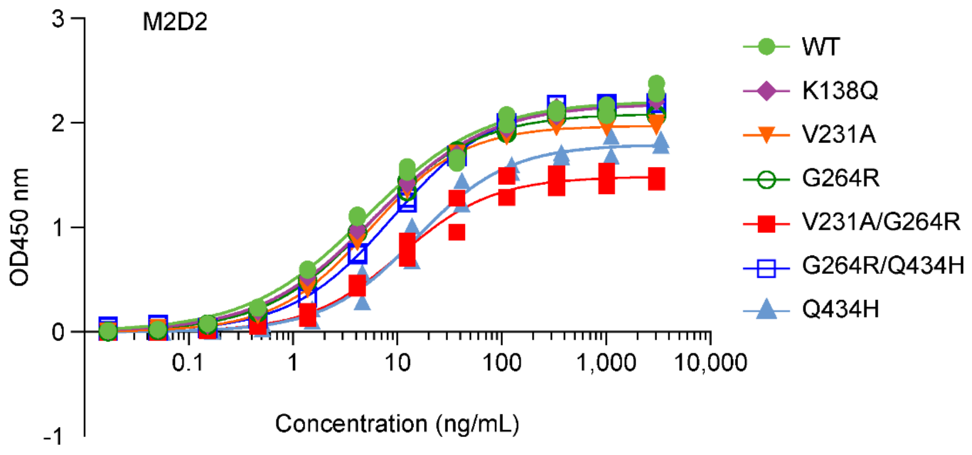

	EC50 (ng/mL)
WT	5.1
K138Q	6.0
V231A	5.5
G264R	5.5
Q434H	15.2
V231A/G264R	10.8
G264R/Q434H	9.1

Supplementary Figure 4. Binding M2D2 (positive control) IgG to unprocessed hMPV PreF and PreF carrying MARM mutants, determined by ELISA with Expi293 cell culture supernatants containing expressed antigens. Error bars indicates the standard deviation of two replicates. Source data are provided as a Source Data file.

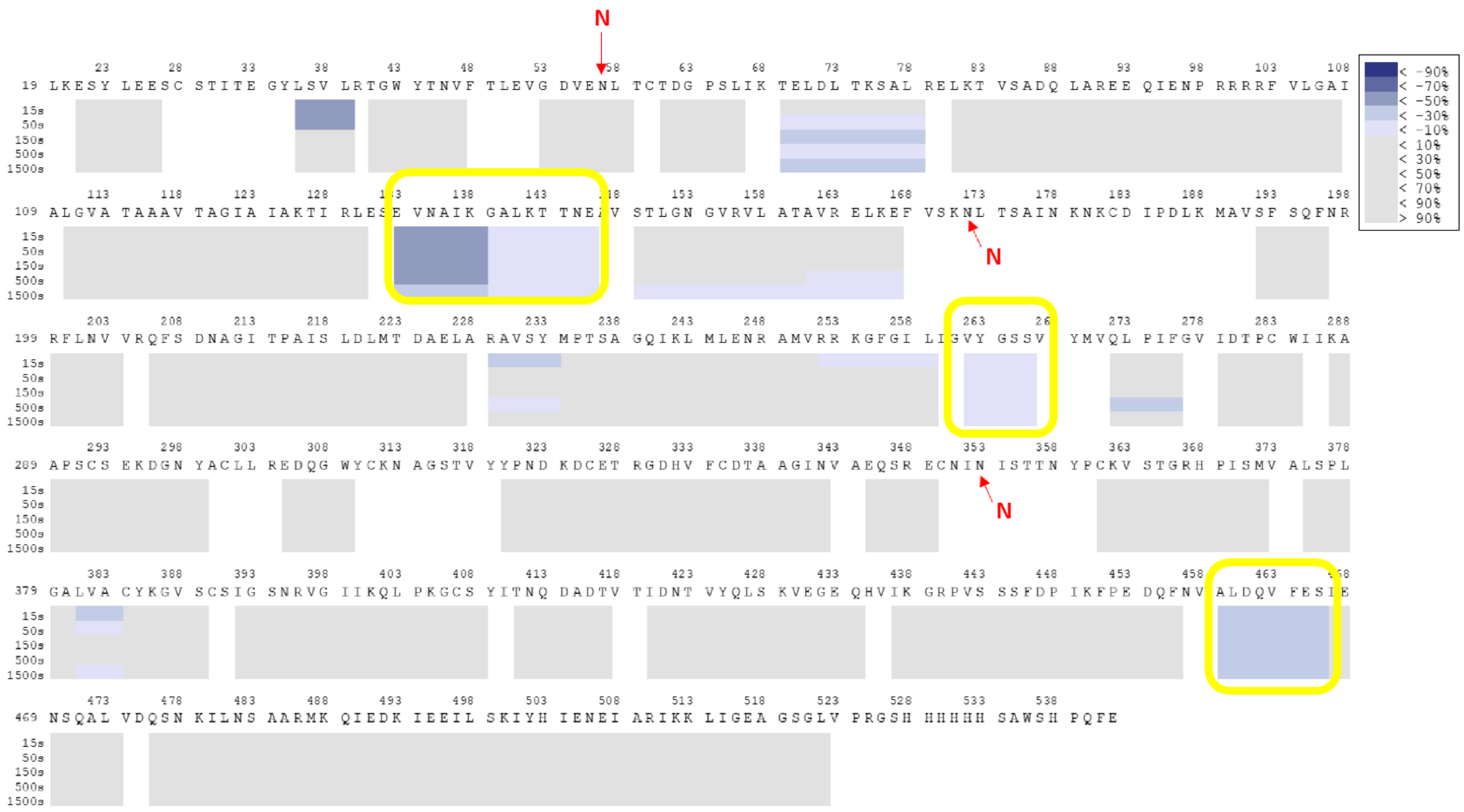

Supplementary Figure 5. Epitope mapping of M4B06 by hydrogen/deuterium-exchange mass spectrometry. Heat map plot showing the difference in deuteration levels of the processed hMPV PreF trimer protein alone compared to the antigen in the presence of the M4B06 monovalent Fab at five time points (15, 50, 150, 500, and 1500 sec). Slower deuterium exchange (highlighted in yellow) indicates regions containing the binding sites. White areas are 'gaps' for which there was no sequence coverage, and thus no HDX-MS information was obtained.

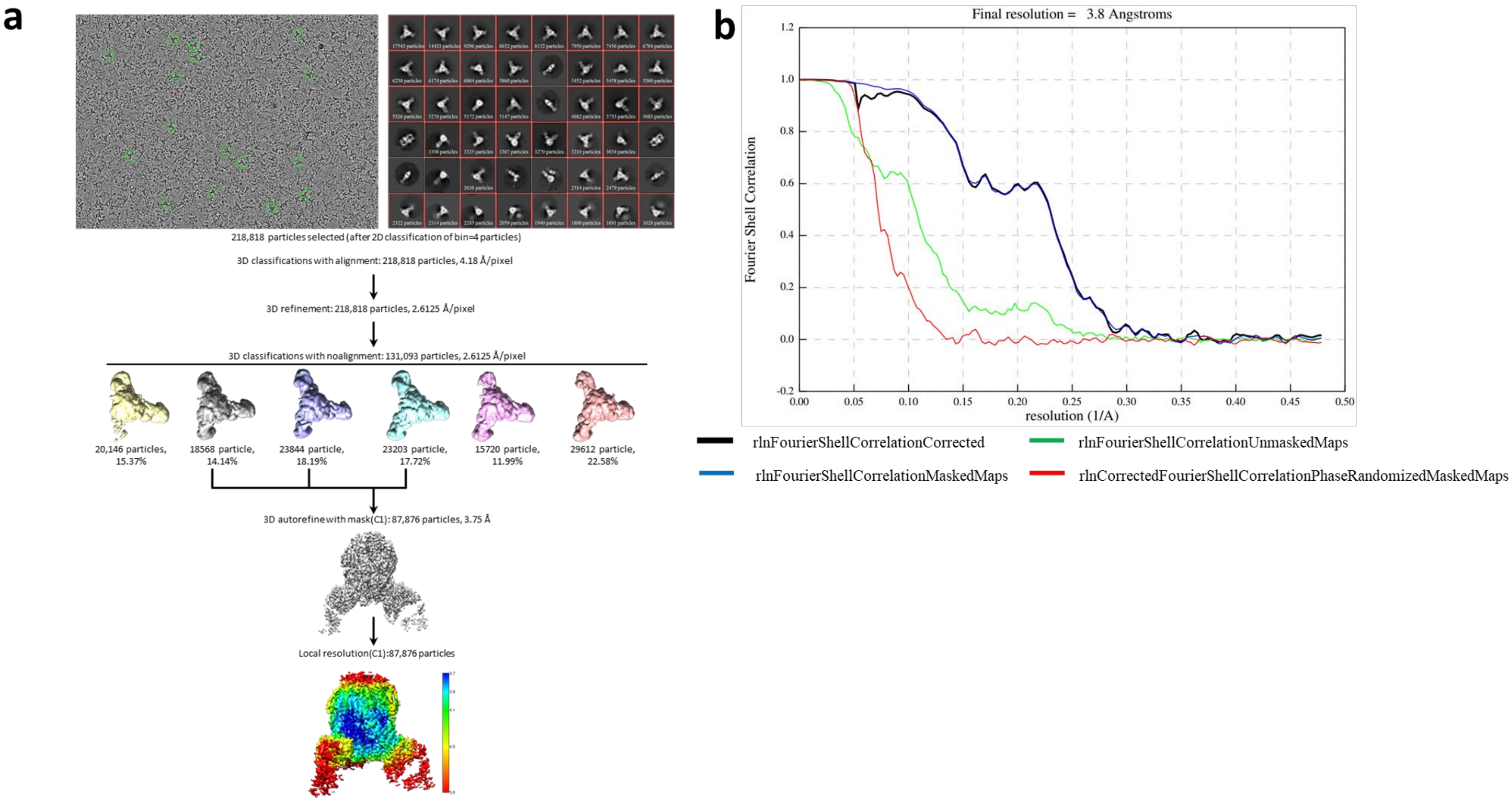

Supplementary Figure 6. CryoEM data of M4B06 Fab in complex with processed hMPV PreF trimer. (**A**) The flowchart of 3D reconstruction the trivalent immune complex (C1 symmetry) to 3.75 Å resolution. (**B**) FSC curve of the 3D reconstruction of the trivalent immune complex with C1 symmetry enforced. It shows that the map has the resolution of 3.75 Å which is the Nyquist frequency with the pixel size of 1.045 Å.

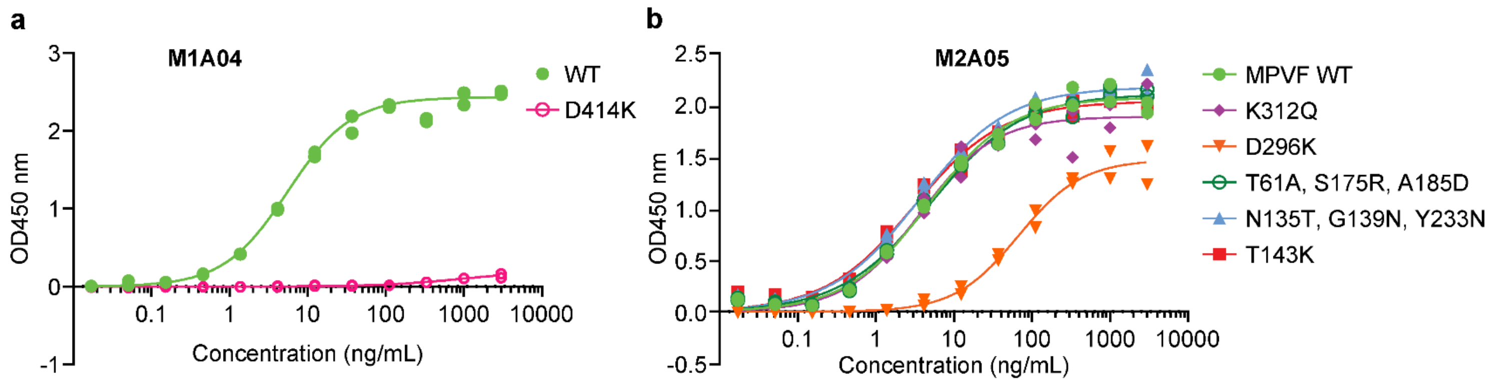

	EC50 (ng/mL)
WT	5.5
D414K	No Binding

	EC50 (ng/mL)
WT	4.7
K312Q	3.6
D296K	67.2
T61A, S175R, A185D	5.1
N135T, G139N, Y233N	3.6
T143K	3.0

Supplementary Figure 7. Binding of M1A04 (A) and M2A05 (B) IgG to unprocessed hMPV PreF and mutants, determined by ELISA with Expi293 cell culture supernatants containing expressed antigens. Error bars indicates the standard deviation of two replicates. Source data are provided as a Source Data file.

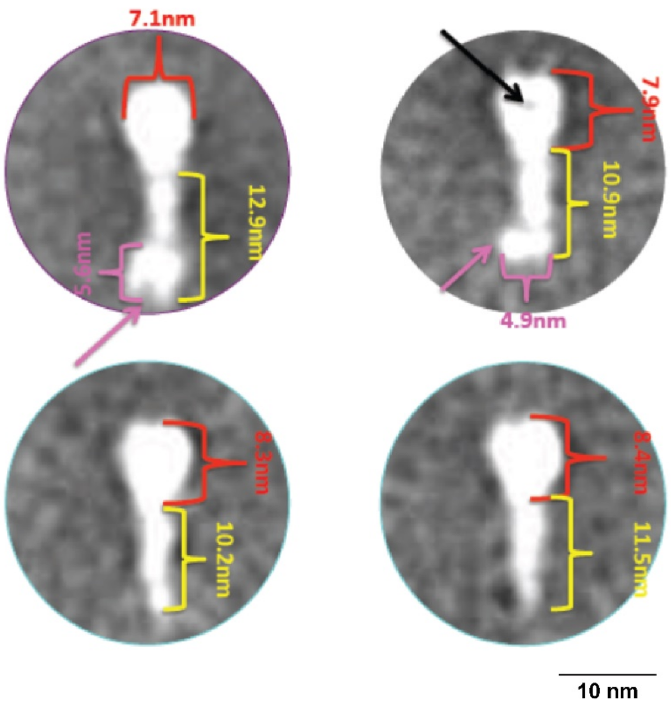

Supplementary Figure 8. Negative staining EM and 2D average of engineered hMPV postF trimer protein. Most class averages show particles with a distinct head portion (red) and an elongated tail portion (yellow). In some averages the tail portion appears longer with a small distinct portion at the end of the tail (magenta). Visible in some averages was an indentation in the 'head' portion of the particle (black arrow). All figures to scale.

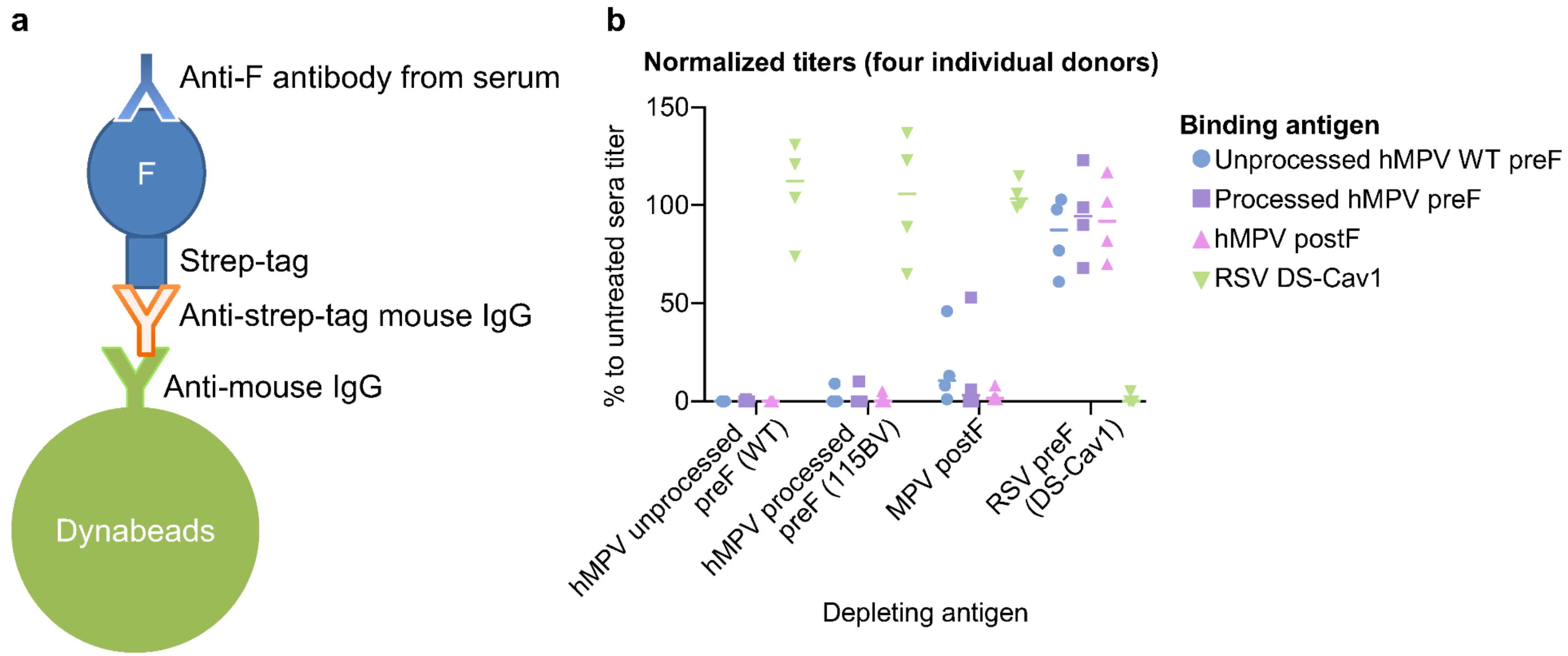

Supplementary Figure 9. Human serum absorption assay with unprocessed hMPV PreF, processed hMPV PreF, hMPV PostF, and RSV PreF trimers. (**A**) Schematic plot showing the format of magnetic bead-based serum absorption assay. (**B**) ELISA titers from four donors after antigen depletion, normalized by titers of un-depleted serum. Middle bars indicate the median. Source data are provided as a Source Data file.

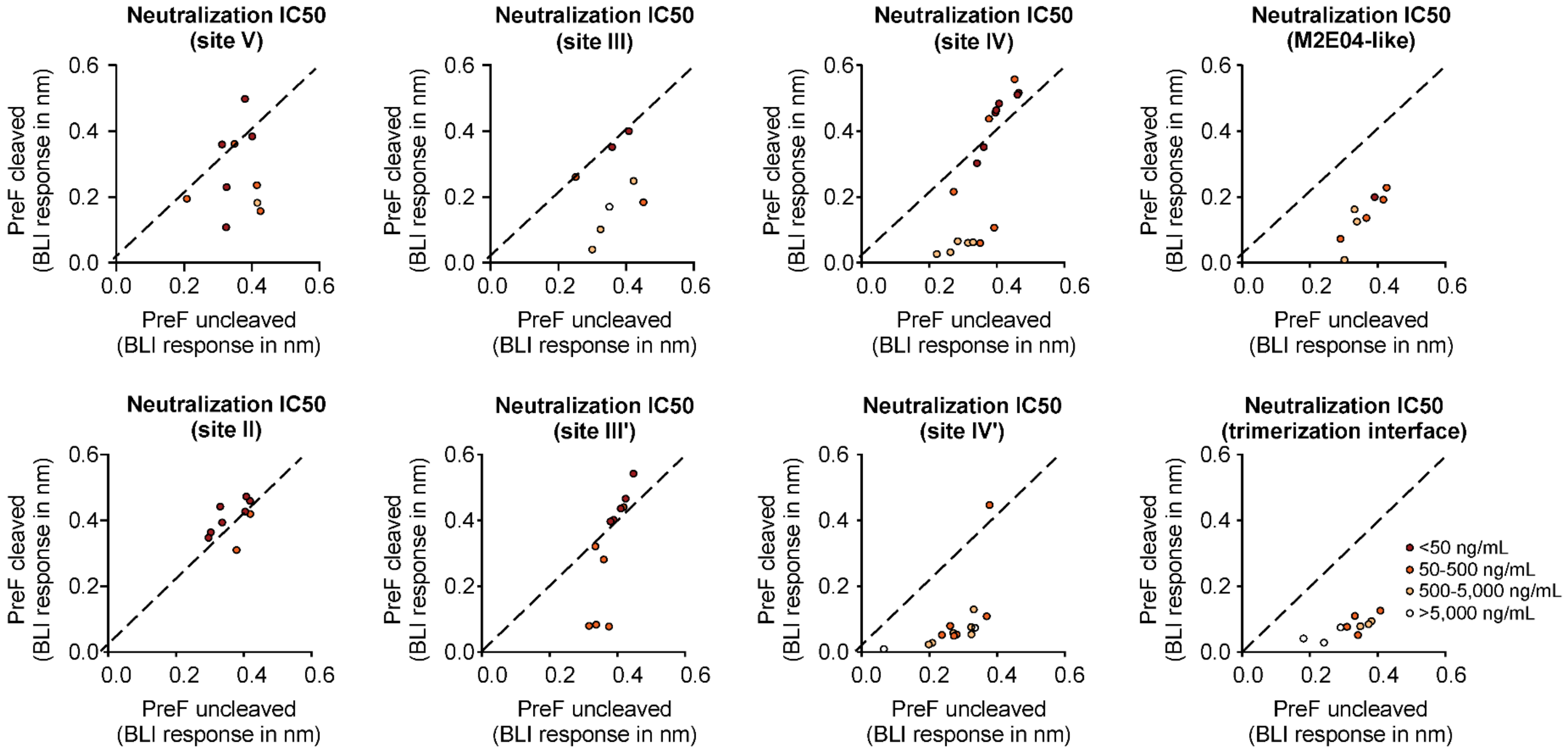

Supplementary Figure 10. BLI binding response of hMPV-F specific mAbs to unprocessed hMPV PreF and processed stabilized hMPV PreF (115BV) antigens, plotted by individual antigenic sites. Every dot represents an isolated hMPV F specific antibody, colored by neutralization potency. For each mAb, the stronger neutralization potency number to hMPV A and B was chosen to determine its coloring. The diagonal dash line indicated binding of unprocessed PreF equals to processed PreF. Source data are provided as a Source Data file.

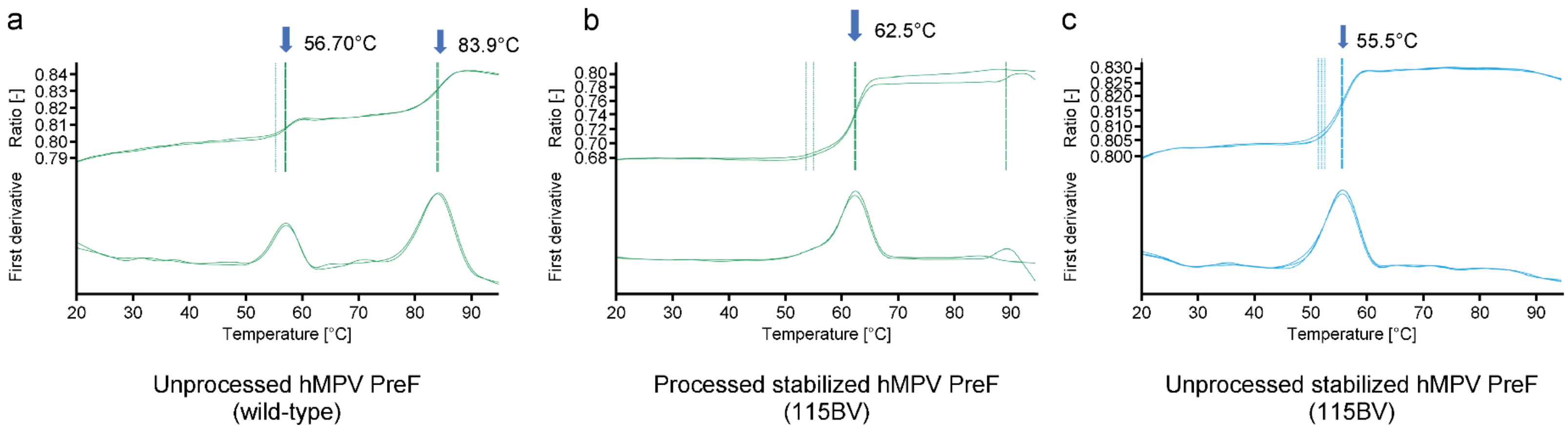

Supplementary Figure 11. Differential scanning fluorimetry (DSF) spectra of (**A**) Unprocessed hMPV PreF protein (1.5 mg/ml); (**B**) Processed stabilized hMPV PreF protein expressed without furin (2.5 mg/ml); (**C**) Unprocessed stabilized hMPV PreF protein expressed without furin cleavage (2.7 mg/ml). Spectra were recorded at a scan rate of 1 °C/min in duplicate. Graphs depict the ratio of the fluorescence between 350 nm and 330 nm (top) as well as the first derivative of this ratio (bottom).

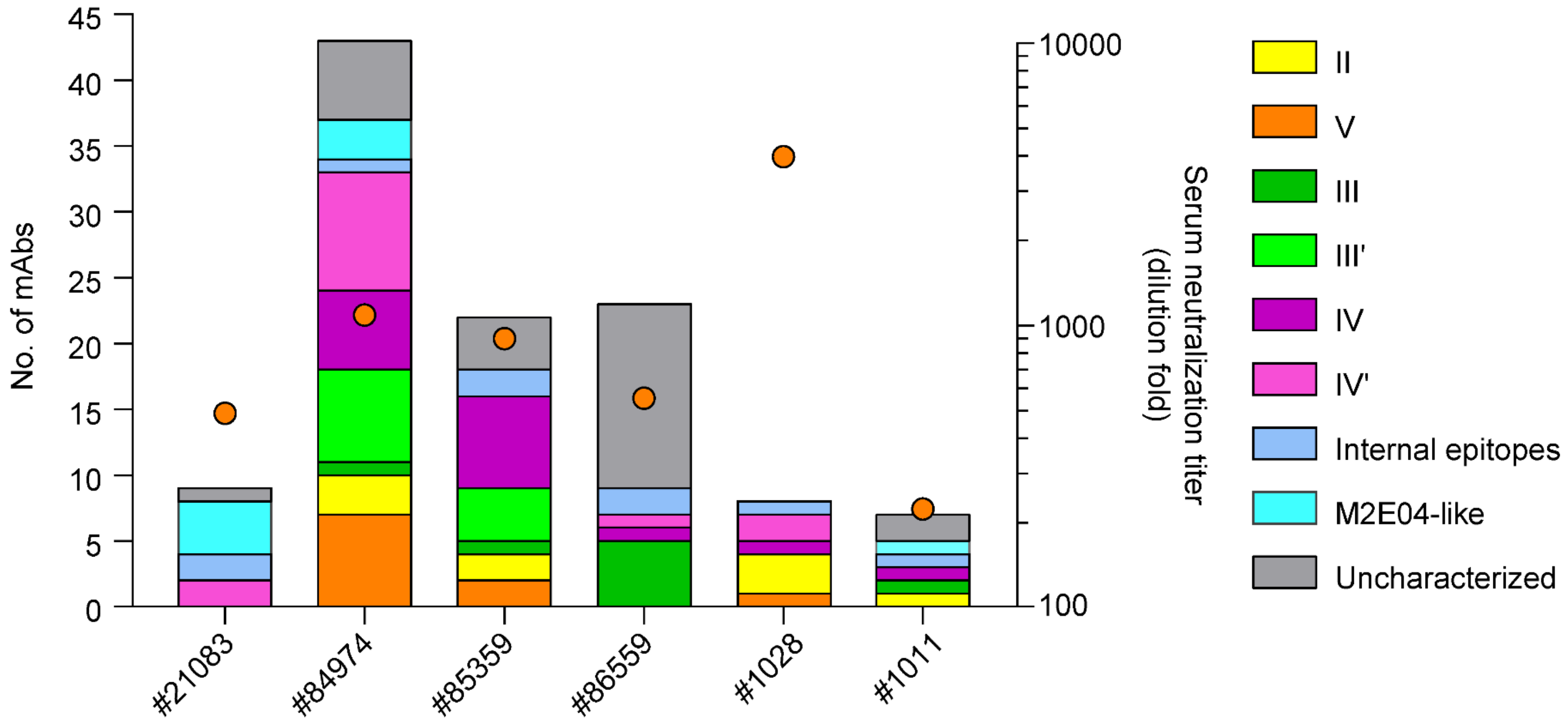

Supplementary Figure 12. Distributions of isolated hMPV-F specific mAbs and their mapped antigenic sites, grouped by donors. Left Y-axis: Number of mAbs discovered from each donor; right Y-axis: serum neutralization titer (fold-dilution) of each donor. Orange dots indicated the neutralization titers of donors' sera in fold dilution. Donor #BSC2 was not included as only one mAb was isolated from this donor. Source data are provided as a Source Data file.

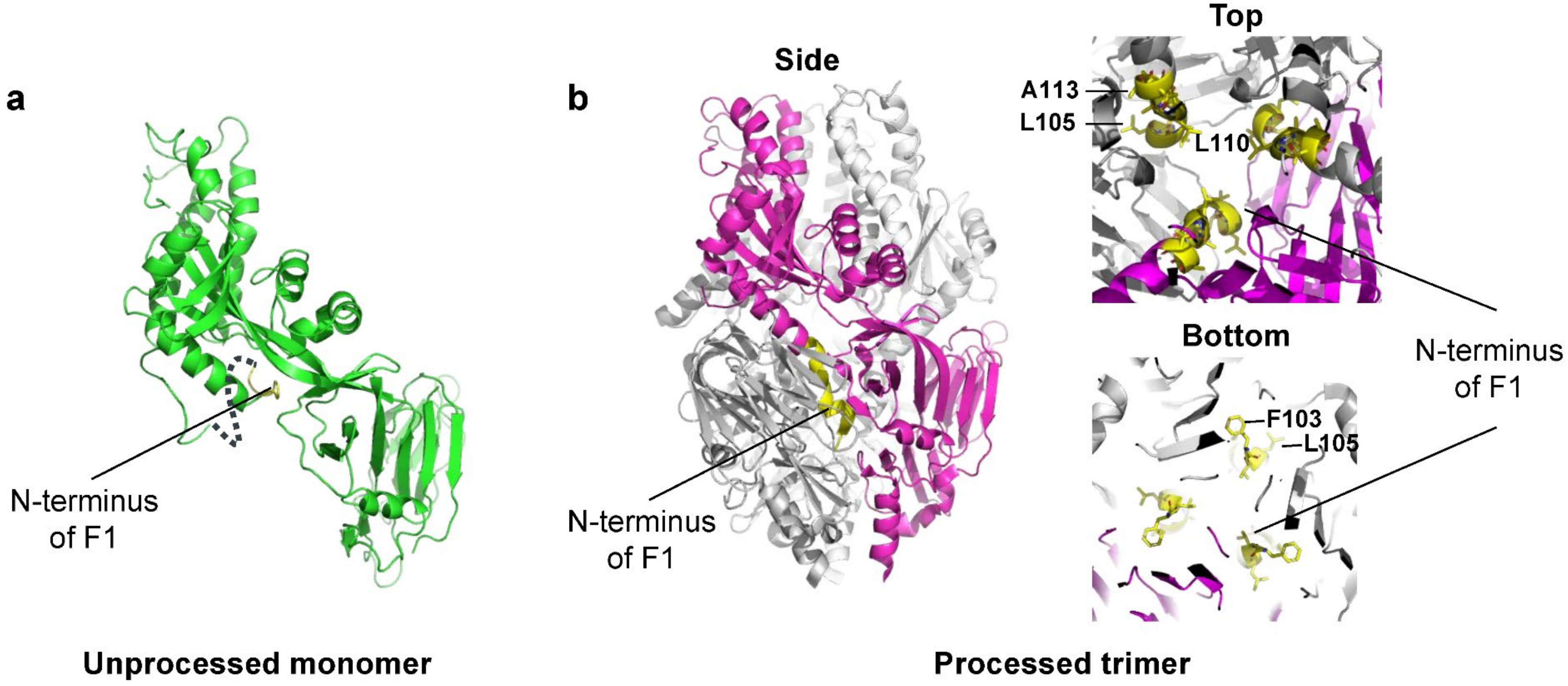

Supplementary Figure 13. Comparison of unprocessed hMPV PreF monomer (A) and processed stabilized hMPV PreF trimer (B). The N-terminus segment of F_1 near cleavage site was labeled in yellow.
