# Supplementary material for: Profiling of hMPV F-specific antibodies isolated from human memory B cells
Source: Nat Commun. 2022 May 10;13:2546. doi: 10.1038/s41467-022-30205-x (PMC9091222; doi:10.1038/s41467-022-30205-x)
Supplement: Supplementary file 3 — Description of Additional Supplementary Files [file 41467_2022_30205_MOESM3_ESM.pdf]

Description of Additional Supplementary Files

File Name: Supplementary Data 1
Description: Information of 113 mAbs reported in the manuscript
