# Supplementary material for: Profiling of hMPV F-specific antibodies isolated from human memory B cells
Source: Nat Commun. 2022 May 10;13:2546. doi: 10.1038/s41467-022-30205-x (PMC9091222; doi:10.1038/s41467-022-30205-x)
Supplement: Supplementary file 5 — Reporting Summary [file 41467_2022_30205_MOESM5_ESM.pdf]

nature research

Corresponding author(s): Kalpit Vora, Zhifeng Chen, Lan Zhang

Last updated by author(s): Jan 5, 2022

Reporting Summary

Nature Research wishes to improve the reproducibility of the work that we publish. This form provides structure for consistency and transparency in reporting. For further information on Nature Research policies, see our Editorial Policies and the Editorial Policy Checklist.

Statistics

For all statistical analyses, confirm that the following items are present in the figure legend, table legend, main text, or Methods section.

n/a	Confirmed	
[ ]	[x]	The exact sample size (*n*) for each experimental group/condition, given as a discrete number and unit of measurement
[ ]	[x]	A statement on whether measurements were taken from distinct samples or whether the same sample was measured repeatedly
[x]	[ ]	The statistical test(s) used AND whether they are one- or two-sided<br>*Only common tests should be described solely by name; describe more complex techniques in the Methods section.*
[x]	[ ]	A description of all covariates tested
[x]	[ ]	A description of any assumptions or corrections, such as tests of normality and adjustment for multiple comparisons
[ ]	[x]	A full description of the statistical parameters including central tendency (e.g. means) or other basic estimates (e.g. regression coefficient) AND variation (e.g. standard deviation) or associated estimates of uncertainty (e.g. confidence intervals)
[x]	[ ]	For null hypothesis testing, the test statistic (e.g. *F*, *t*, *r*) with confidence intervals, effect sizes, degrees of freedom and *P* value noted<br>*Give P values as exact values whenever suitable.*
[x]	[ ]	For Bayesian analysis, information on the choice of priors and Markov chain Monte Carlo settings
[x]	[ ]	For hierarchical and complex designs, identification of the appropriate level for tests and full reporting of outcomes
[x]	[ ]	Estimates of effect sizes (e.g. Cohen's *d*, Pearson's *r*), indicating how they were calculated

Our web collection on statistics for biologists contains articles on many of the points above.

Software and code

Policy information about availability of computer code

Data collection

SONY 800S Cell Sorter (FACS)
Illumina MiSeq (NGS)
Ensight plate reader, Perkin Elmer (neutralization assays)
FEI Titan Krios 300KV electron microscope, FEI Tecnai T12 electron microscope (EM)
Octet Red 96e (BLI)
Perkin Elmer Victor-Multi label counter (ELISA)
ThermoScientific LTQ-XL Orbitrap mass spectrometer (HDX)
Prometheus NT.48 instrument (DSF)

Data analysis

Graphpad prism 8 (ELISA, neutralization, BLI)
Octet software DataAcquisition version 8, ForteBio (BLI)
SONY Cell Sorter Software, version 2.1.5
Proteome Discoverer 1.4 software, Thermo Fisher Scientific and HDExaminer software v1.4(HDX)
PyMOL 1.7.05 (Structural visualization)
FastQC v0.11.2; PANDAseq v2.10; IgBLAST v1.9.0 61 and tblastn (v2.2.29) in the BLAST+ suite; Change-O v0.3.12 (sequence analysis)
RStudio 1.2.5033 (epitope binning data visualization)
Negative staining was analyzed by NanoImagine (Leginon, CTFFIND4, Xmipp)
CryoEM data was analyzed by Biortus (SerialEM software, RELION 3.0, Gctf, Chimera)

For manuscripts utilizing custom algorithms or software that are central to the research but not yet described in published literature, software must be made available to editors and reviewers. We strongly encourage code deposition in a community repository (e.g. GitHub). See the Nature Research guidelines for submitting code & software for further information.

Data

Policy information about availability of data

All manuscripts must include a data availability statement. This statement should provide the following information, where applicable:

- Accession codes, unique identifiers, or web links for publicly available datasets
- A list of figures that have associated raw data
- A description of any restrictions on data availability

The authors declare that the data supporting the findings of this study are available within the main and supplemental materials. All data are available from the corresponding author upon reasonable request.

Field-specific reporting

Please select the one below that is the best fit for your research. If you are not sure, read the appropriate sections before making your selection.

[x] Life sciences [ ] Behavioural & social sciences [ ] Ecological, evolutionary & environmental sciences

For a reference copy of the document with all sections, see nature.com/documents/nr-reporting-summary-flat.pdf

Life sciences study design

All studies must disclose on these points even when the disclosure is negative.

Sample size	No animal or human studies are involved in this report. No sample size calculation was performed to design the study. Each individual sample was screened for serum hMPV neutralization titers and the antibodies isolated from memory B cells of a given individual are reported in this study. These antibodies are representative of the repertoire of each individual.
Data exclusions	No data points were excluded
Replication	Individual recombinant antibodies were tested in binding assays and neutralization assays in duplicate or triplicate and experiments were repeated as detailed in the figure legends for each experiments. As indicated experiments could be repeated with similar results. Some experiments were repeated by different operators and in different laboratories. All data generated with these antibodies are reported in this manuscript.
Randomization	No randomization was used in this study.Randomization is not applicable.
Blinding	Blinding is not relevant for this study because it is not a case control study.

Reporting for specific materials, systems and methods

We require information from authors about some types of materials, experimental systems and methods used in many studies. Here, indicate whether each material, system or method listed is relevant to your study. If you are not sure if a list item applies to your research, read the appropriate section before selecting a response.

Materials & experimental systems

n/a	Involved in the study	
[ ]	[x]	Antibodies
[ ]	[x]	Eukaryotic cell lines
[x]	[ ]	Palaeontology and archaeology
[x]	[ ]	Animals and other organisms
[x]	[ ]	Human research participants
[x]	[ ]	Clinical data
[x]	[ ]	Dual use research of concern

Methods

n/a	Involved in the study	
[x]	[ ]	ChIP-seq
[ ]	[x]	Flow cytometry
[x]	[ ]	MRI-based neuroimaging

Antibodies

Antibodies used	CD3 PEcy7 BD Biosciences catalog 563423 (1:60), IgG APC BD Biosciences catalog 550931 (1:15), CD19 FITC BD Biosciences catalog 555415 (1:15)<br>mouse anti-hMPV mAb clone 132 (EMD Millipore MAB80124)<br>anti-mouse IgG Alexa 488 conjugated secondary antibody (Invitrogen #A11017, 1:500)<br>HRP-conjugated goat anti-human IgG (Southern Biotech #2040-05, 1:2,000)<br>Anti-RSV F murine antibody, clone 143-F3-B138, in house generated from our own hybridoma<br>Anti-RSV N murine antibody, clone 34C9, in house generated from our own hybridoma

Validation

Anti-RSV F and anti-RSV N clones generated in-house were derived from mice and tested for reactivity against the associated RSV protein in immunoassay prior to experiments with a irrelevant antibody as a negative control.
All other antibodies were purchased from reputable vendors with technical data sheets.

Eukaryotic cell lines

Policy information about cell lines

Cell line source(s)

LLC-MK2 (ATCC Cat#CCL-7.1), HEp-2 (Human Epithelial Type 2, ATCC Cat#CCL-23), Vero (ATCC Cat#CCL-81)

Authentication

The cell lines came from reputable vendors with certificate of analysis. No further authentication was performed. However, the behavior and morphology of the cells is routinely monitored.

Mycoplasma contamination

All cell lines used were tested and were mycoplasma free.

Commonly misidentified lines
(See ICLAC register)

HEp-2 is listed on the ICLAC list version 9. This cell line was used because RSV grows in these cells.

Flow Cytometry

Plots

Confirm that:

- [x] The axis labels state the marker and fluorochrome used (e.g. CD4-FITC).
- [x] The axis scales are clearly visible. Include numbers along axes only for bottom left plot of group (a 'group' is an analysis of identical markers).
- [x] All plots are contour plots with outliers or pseudocolor plots.
- [x] A numerical value for number of cells or percentage (with statistics) is provided.

Methodology

Sample preparation

PBMC from selected donors were isolated from whole blood on the same day as blood collection, using a Accuspin tube (AccuspinTM System-Histopaque®-1077 (Sigma Cat#A6929). Cells where then frozen in 90% Fetal calf serum with 10% DMSO, and stored in liquid nitrogen until thawed for experiments. Cryopreserved PBMCs were thawed on the day of sorting and the B cell population was enriched using the EasySepTM Human B-cell Enrichment Kit (Stemcell Technologies). Next, B cells were stained with biotinylated or Alexa 647 tagged F antigens and then followed by staining with a panel of monoclonal antibodies including anti-CD3 mAb-PE-CyTm 7 (BD Biosciences Cat#563423, 1:60 dilution), anti-CD19-FITC (BD Biosciences Cat#555415, 1:15 dilution), anti-human IgG-APC (BD Biosciences Cat#550931, 1:15 dilution), and PE-streptavidin. CD3−/CD19+/IgG+/F+ cells were sorted with a SONY 800S Cell Sorter in single cell mode into a 96-well plate.

Instrument

BD Sony S800 cell sorter

Software

Cell Sorter Software, version 2.1.5

Cell population abundance

The Sony SH800 sorter was calibrated each day prior to use. Additionally, to check the sorting purity, a more abundant population (i,e. CD3-IgG+) was sorted and reacquired to evaluate purity and function of the instrument. The target population (antigen specific memory B cells) were rare events and thus could not be sorted and then re-run for purity. From the downstream cloning process it was apparent that single B cells were sorted.

Gating strategy

Lymphocytes were gated based on size and granularity (FSC vs BSC), followed by Forward scatter height vs area to exclude doublets. Next , cells were gated on CD3-.(Note that this was the majority of the cells because a B cell magnetic bead kit was used prior to staining the cells. This gate was set based on running a sample that was not pre-enriched to determine where the CD3+ population was). Next, cells were gated on CD19+IgG+ population. The placement of that gate is apparent when viewing the pseudocolor dot plots in terms of the IgG positive and negative areas. Next, we placed a gate on the cells binding to hMPV-PE. The placement of this gate was determined by running a negative control during set-up (cells that were not incubated with biotinylated hMPV, and stained only with the streptdavidin PE).

- [x] Tick this box to confirm that a figure exemplifying the gating strategy is provided in the Supplementary Information.
